# High Level Constitutive Expression of Luciferase Reporter by *lsd90* Promoter in Fission Yeast

**DOI:** 10.1371/journal.pone.0101201

**Published:** 2014-07-07

**Authors:** Hemant Kumar Verma, Poonam Shukla, Md. Alfatah, Asheesh Kumar Khare, Udita Upadhyay, Kaliannan Ganesan, Jagmohan Singh

**Affiliations:** Institute of Microbial Technology, Chandigarh, India; Newcastle University, United Kingdom

## Abstract

Because of a large number of molecular similarities with higher eukaryotes, the fission yeast *Schizosaccharomyces pombe* has been considered a potentially ideal host for expressing human proteins having therapeutic and pharmaceutical applications. However, efforts in this direction are hampered by lack of a strong promoter. Here, we report the isolation and characterization of a strong, constitutive promoter from *S. pombe*. A new expression vector was constructed by cloning the putative promoter region of the *lsd90* gene (earlier reported to be strongly induced by heat stress) into a previously reported high copy number vector pJH5, which contained an ARS element corresponding to the *mat2P* flanking region and a truncated *URA3m* selectable marker. The resulting vector was used to study and compare the level of expression of the luciferase reporter with that achieved with the known vectors containing regulatable promoter *nmt1* and the strong constitutive promoter *adh1* in *S. pombe* and the methanol-inducible *AOX1* promoter in *Pichia pastoris*. Following growth in standard media the new vector containing the putative *lsd90* promoter provided constitutive expression of luciferase, at a level, which was 19-, 39- and 10-fold higher than that achieved with *nmt1*, *adh1* and *AOX1* promoters, respectively. These results indicate a great potential of the new *lsd90* promoter-based vector for commercial scale expression of therapeutic proteins in *S. pombe*.

## Introduction

Among various yeast species, the methylotrophic yeast *Pichia pastoris* has emerged as a useful expression host for commercial scale production of therapeutic proteins. Reasons behind popularity of the *Pichia* system are availability of *AOX1* promoter, a strong methanol-inducible promoter of alcohol oxidase I and ease of growth to very high cell density in an inexpensive, non-complex and chemically defined medium [1]–[4]. However, despite these advantages, there are serious issues regarding safety aspects of *Pichia* expression system, which uses inflammable methanol for induction; methanol is added after every 24 hrs of growth phase and maximum expression is achieved only after 4–6 days of induction. Large-scale fermentation using considerable amounts of methanol necessitates suitable precautions and special fermenter design to ensure safety. In addition, disposal of toxic waste containing methanol is of grave environmental concern.

Among other well-characterized yeast species, the fission yeast *Schizosaccharomyces pombe* has been recognized as an excellent model system for understanding biological phenomena at the cellular and molecular level [5], [6]. In comparison with the budding yeast *Saccharomyces cerevisiae*, *S. pombe* shares greater level of similarity with higher eukaryotes. For example, the conservation of splicing machinery which allow human intron-containing genes to be spliced in *S. pombe*, the complexity of replication origins and centromeric regions, gene regulatory mechanisms, existence of similar components of RNAi and heterochromatin mechineries, presence of intact Golgi apparatus, etc. [5], [6]. Because of these similarities, *S. pombe* is considered as an attractive host for the production of proteins of eukayotic origin [5], [6]. Expression vectors for high-level expression in *S. pombe* have been developed and many foreign proteins successfully expressed [5], [6]. However, *S. pombe* has lagged behind the *P. pastoris* expression system mainly because of lack of strong promoters. Among them, *nmt1*, the strongest known regulatable promoter, is repressed by thiamine and has been widely used for heterologous gene expression [5], [7]. To further achieve graded levels of expression, two additional weakened versions of the *nmt1* promoter, denoted as *nmt41* and *nmt81*, provide medium and low levels of expression, respectively [8]. Use of mammalian viral promoters has also been reported in *S. pombe* [9]. However, these promoters are not suitable for commercial scale expression because they do not achieve expression levels comparable to *P. pastoris* and are not user-friendly. In our experience, *nmt1* promoter yielded only moderate level of expression of a therapeutic protein, indicating inadequacy of *nmt1* for commercial scale expression of proteins [10].

Several other promoters have been used for regulated expression of heterologous genes in *S. pombe* but the expression level was even lower than *nmt1* promoter [11]–[14]. *adh1*, the strongest known constitutive promoter can provide expression levels up to 0.5–2.5% of total protein [15], [16]. However, the maximum β-galactosidase activity achieved under the *adh1* promoter was almost half of that achieved under the *nmt1* promoter [17]. Moreover, constitutive expression of the heterologous gene may sometimes be toxic to the host cell, although it depends on the nature of the protein to be expressed. Thus, isolation of new stronger promoters in *S. pombe* that can be employed at industrial scale with minimum effort and inexpensive media is an area requiring greater systematic and sustained effort.

Luciferase has often been used as a reporter gene in bacterial and mammalian cells. There are very few reports describing the use of luciferase as a reporter in *S. cerevisiae* [18], [19]. Firefly luciferase (Fluc) has been used to study transcriptional and translational fidelity in yeast [20]. Recently, Fluc protein was used as a marker to evaluate the efficacy of the vectors with high copy number and mitotic stability for high-level expression of heterologous proteins in *Hansenula polymorpha* [21]. The Fluc reporter has also been used as a biosensor for screening compounds toxic to eukaryotes [22]. Fluc has proved highly effective as a reporter gene, since the luciferase assays are extremely sensitive, rapid, reproducible and easy to perform [19].

Earlier, we constructed a new high copy vector pJH5 containing the *mat2P*-linked ARS element from *S. pombe* and a truncated *URA3m* gene from *S. cerevisiae* as a selectable marker, which occurs at very high copy number, ∼200 copies/cell [23]. In this study, we used this vector as backbone for cloning the new promoter. We initiated screening of new promoters based on the published microarray data [24] and measured the activity of putative promoter elements using luciferase as the reporter gene. The resulting vector provides constitutive expression of luciferase reporter which is considerably higher than not only the expression achievable with the vectors containing *nmt1* and *adh1* promoters in *S. pombe* but also the *AOX1* promoter based vector in *P. pastoris*.

## Results

### Construction of expression vectors

**Figure 1:**
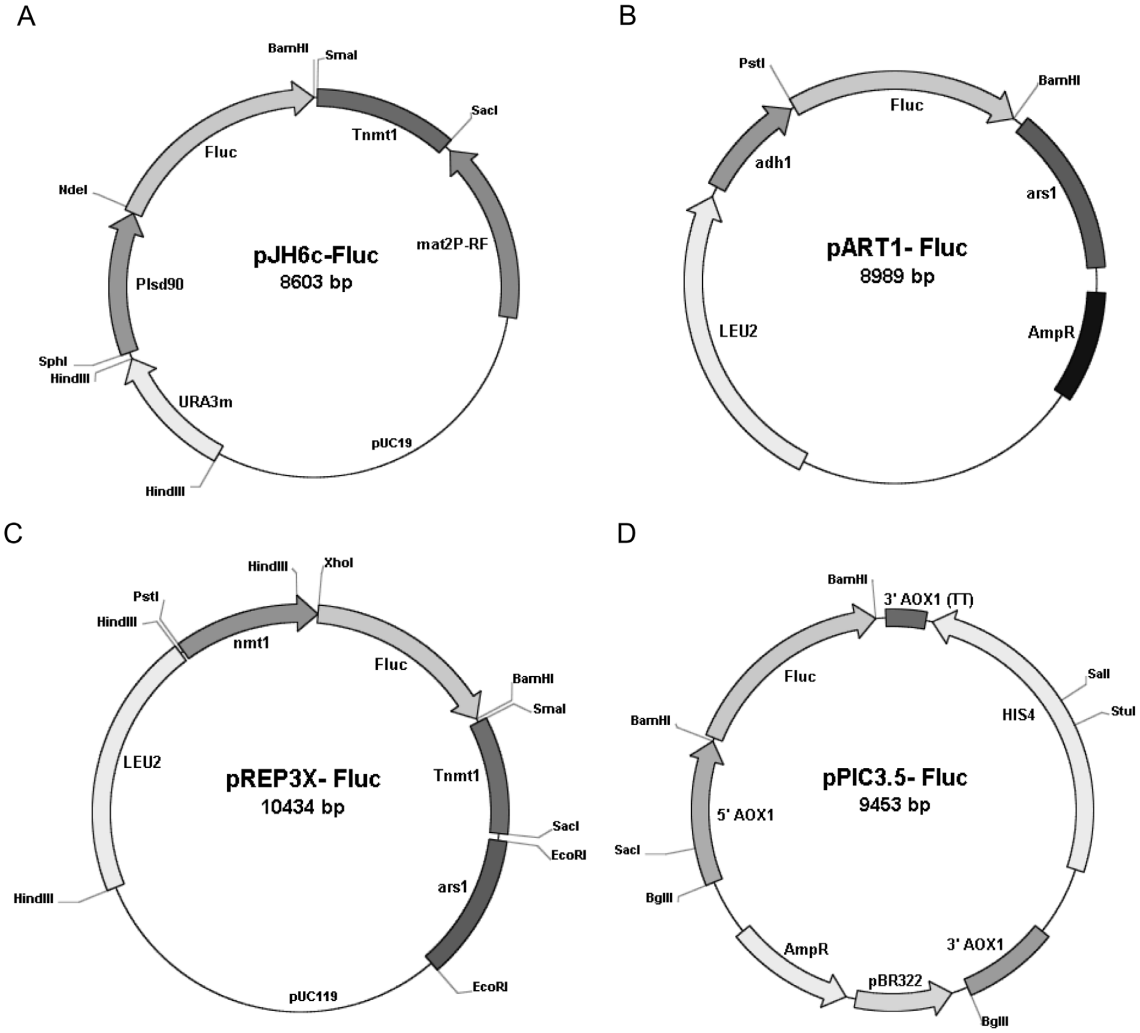
Schematic diagram of the Fluc-expression vectors used in this study. Restriction map of Fluc-expression vectors; (A) pJH6c-Fluc, (B) pART1-Fluc, (C) pREP3X-Fluc and (D) pPIC3.5-Fluc. Approximately 1.0 kb upstream region of gene *SPAC1F8.02C/lsd90* of *S. pombe* was PCR amplified and inserted into the plasmid pJH5 at SphI/NdeI sites as promoter *Plsd90* respectively. The resulting vectors were designated as pJH6c (A). The strategy of *Fluc* cloning is described in the methods section.

To search for new stronger and regulatable promoters, we screened the published DNA microarray data of global transcriptional responses of *S. pombe* to various environmental stresses [24]. The published data showed that genes *SPAC1F8.02C* and *SPBC24C6.09C* showed maximum induction of RNA levels (260- and 108-fold, respectively) when exposed to 0.5 mM H_2_O_2_, while *lsd90/SPBC16E9.16C* showed maximum induction (117-fold) upon shifting the culture from 30°C to 39°C. Accordingly, approximately 1.0 to 1.5 kb upstream regions of these genes were PCR amplified as putative promoters and cloned into the vector pJH5 [23] to obtain new expression vectors pJH6a, pJH6b (not shown) and pJH6c (Figure 1), respectively. To assess the promoter efficiency, *Fluc* reporter gene was cloned downstream of these promoters (Figure 1). For a comparative study with existing expression systems, *Fluc* gene was also cloned downstream of *adh1* (vector pART1) and *nmt1* promoter (vector pREP3X) of *S. pombe* and *AOX1* promoter (vector pPIC3.5) of *P. pastoris* (Figure 1).

#### Strong, constitutive expression of Fluc-gene by putative promoter region of *lsd90* gene

The putative expression vectors were transformed into the *S. pombe* strain SPJ25. Transformants were grown in synthetic medium and expression of the luciferase reporter gene in response to various stresses [24] was studied. However, contrary to expectation based on the published microarray data [24] very low Fluc expression was observed in cells expressing *Fluc* gene under the control of the *Psp1* and *Psp2* promoters (1.7×10^−18^ moles/100 ng and 2.3×10^−18^ moles/100 ng, respectively). Subjecting the cells to oxidative stress also showed no effect (data not shown). The discrepancy of these results from the published microarray data is surprising. This may be due to lack of some further upstream regulatory sequences or presence of negative regulatory sequences inhibiting the induction of the existing promoter elements in the putative promoter region in response to oxidative stress. Alternatively, growth conditions like media composition may account for the difference from the published results [24].

**Figure 2:**
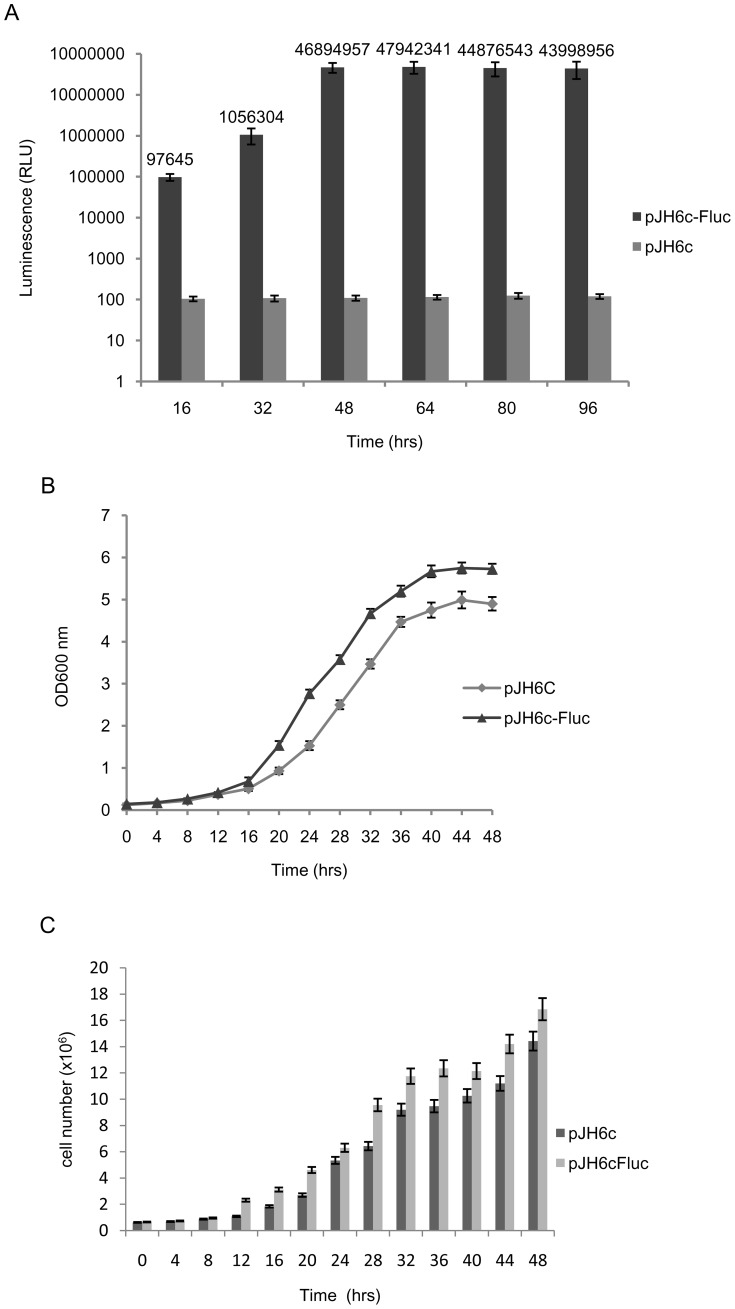
Constitutive expression of the Firefly luciferase (*Fluc*) under lsd90 promoter. Cultures of strain *SPJ25* harboring the vector constructs pJH6c-Fluc and pJH6c were grown in selective media (PMA ura^−^) at 30°C and 200 rpm. Aliquots were taken at the indicated time points and subjected to protein extraction. (A) Luciferase activity was determined using the Luciferase Assay System (Promega, USA). Assays were done in triplicate and the average luminescence values were plotted against the indicated time points. (B) Graph showing growth kinetics of cultures up to early stationary phase. (C) The histogram shows the cell density in Cells/ml at the indicated time points of culture.

In contrast, the vector pH6c-Fluc containing the *lsd90* promoter (*Plsd90*) provided considerably higher level of Fluc expression, which showed increase with cell density, reaching the highest level of 2.4×10^−14^ moles [(∼2.4 ng)/100 ng protein] during early stationary phase (48 hrs). Thereafter, expression level remained almost constant, with a slight increase after 64 hrs [2.5×10^−14^ moles (∼2.5 ng)/100 ng protein], which amounted to 2.5% of total cellular protein (Figure 2A). Furthermore, contrary to the published microarray data [24] exposure of cells to heat stress did not cause a further increase in the level of expression of Fluc. Thus, the putative *lsd90* promoter fragment appears to elicit constitutive expression of luciferase (not shown).

It is possible that constitutive and high level of heterologous gene expression may exert a metabolic load thus leading to slow growth of host cells compared to cells with vector alone. Surprisingly, cells expressing Fluc gene under *lsd90* promoter showed a faster growth rate and achieved higher cell density than those containing the control vector (Figure 2B).

The increase in OD_600_ may also occur indirectly; for example, it may occur due to an increase in cell size. Therefore, we counted the cell density by counting the number of cells/ml by haemocytometry. The results closely parallel the data shown in Figure 2B, indicating a faster growth rate and higher cell density in case of cells harboring the recombinant vector pJH6c-Fluc in comparison with those harboring the control vector pJH6c (Figure 2C).

### Comparison of new expression vector with existing systems of *S. pombe*

**Figure 3:**
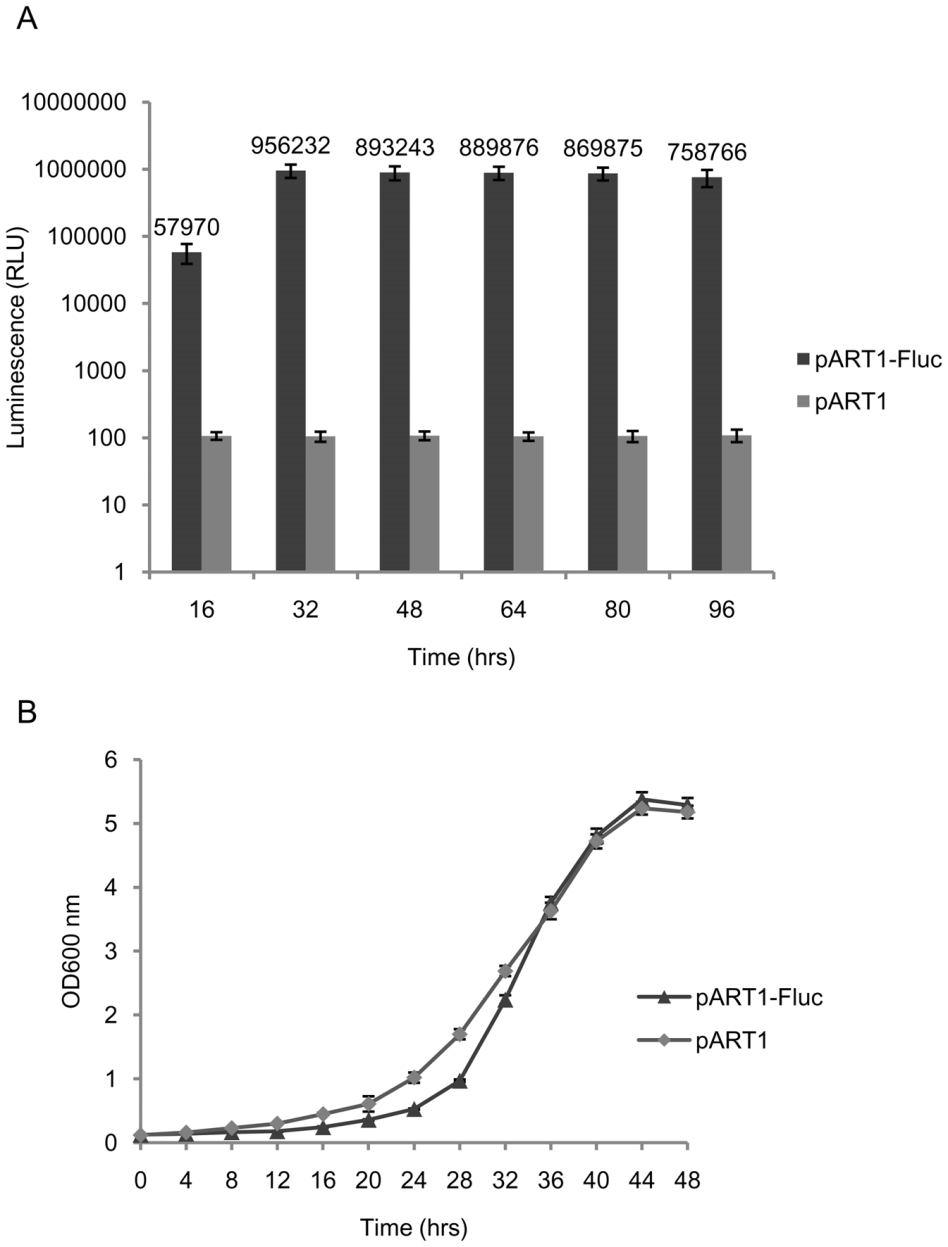
Time course of *Fluc*-expression under control of the *adh1* promoter of *S. pombe*. Cultures of strain *SPJ25* harboring the vector constructs pART1-Fluc and pART1 were grown in selective media (PMA leu^−^) at 30°C and 200 rpm. (A) Luciferase activity measured in RLU and (B) growth kinetics of cultures.

To compare the suitability of the new *lsd90* promoter containing vector as an alternative expression vector, kinetics of expression of luciferase under the control of *adh1* and *nmt1* promoters was also studied. Luciferase expression under *adh1* promoter (in vector pART1) showed an increase up to log phase of growth with maximum level of 6.4×10^−16^ moles [(∼0.064 ng)/100 ng protein] after 32 hrs of growth. A slight decline (5.1×10^−16^ moles/100 ng protein) was observed at 96 hrs of growth, probably due to decrease in the growth rate upon nutrient depletion (Figure 3A). Importantly, the maximum level of luciferase expression achieved with vector pART1-Fluc under the *adh1* promoter was ∼39-fold lower as compared to that achieved with the vector pJH6c-Fluc under *lsd90* promoter (Figure 2A; Table 1).

**Table 1:** Comparison of levels of expression of luciferase using different expression systems.

Vector	Promoter	Maximum Luciferase Expression Level (moles/100 ng)	Relative level w.r.t. *Plsd90*
pJH6c-Fluc	*lsd90*	2.5×10^−14^	1
pART1-Fluc	*adh1*	6.4×10^−16^	1/39
pREP3X-Fluc	*nmt1*	1.3×10^−15^	1/19
pPIC3.5-Fluc	*AOX1*	2.5×10^−15^	1/10

**Figure 4:**
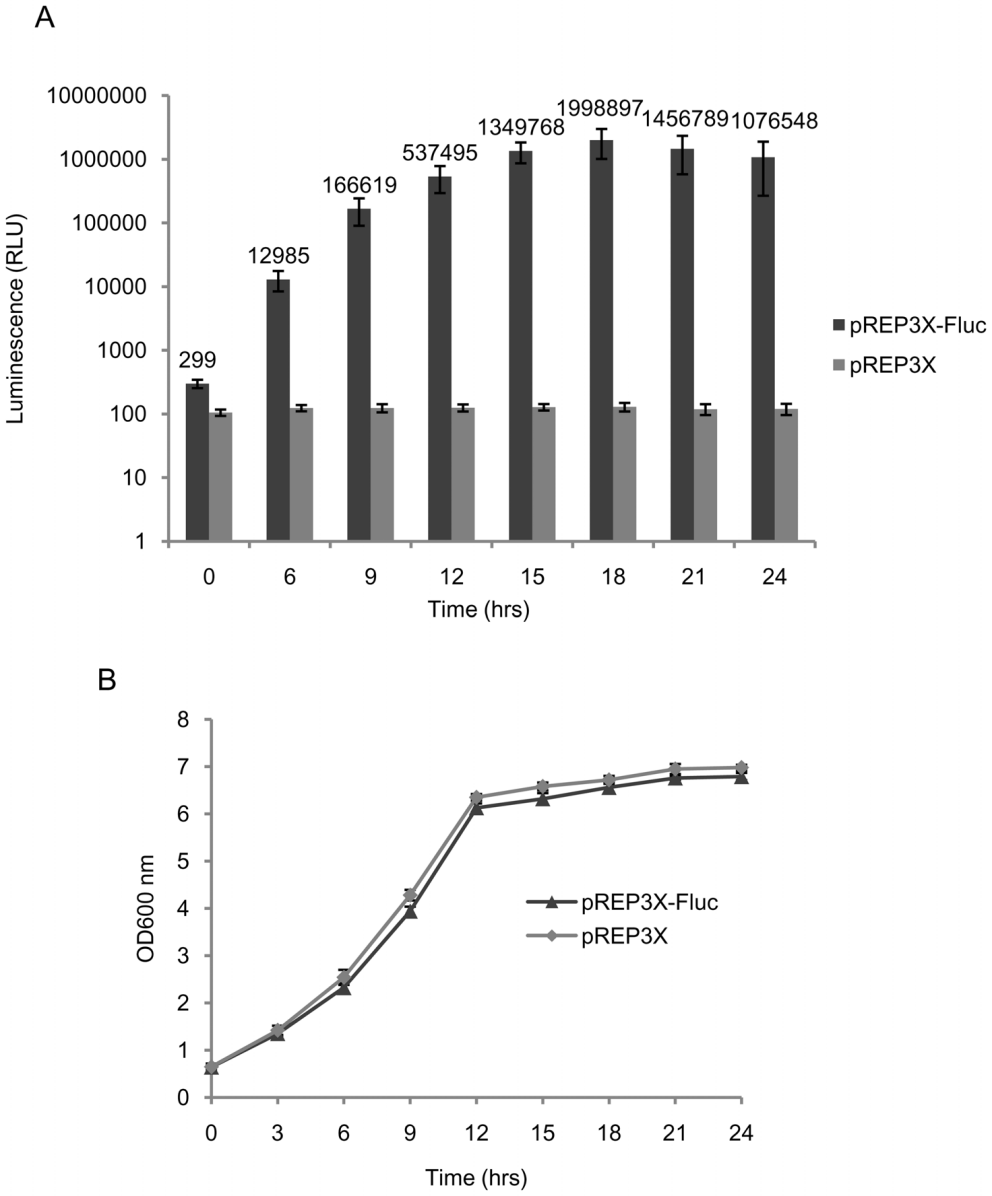
Time course of Fluc-expression under control of the *nmt1* promoter of *S. pombe*. Cultures of strain *SPJ25* harboring the vector constructs pREP3X-Fluc and pREP3X were grown in selective media (PMA leu^−^) at 30°C and 200 rpm. Initially the cultures were grown in medium containing thiamine and then sub-cultured in medium lacking thiamine for the indicated time points. (A) Luciferase activity measured in RLU and (B) growth kinetics of cultures.

Similar, when expressed under the control of the *nmt1* promoter (vector pREP3X), the maximum level of luciferase expression of 1.3×10^−15^ moles [(∼0.13 ng)/100 ng protein] was recorded after 18 hrs of induction (Figure 4A). This level was almost 19-fold lower as compared to maximum expression level with vector pJH6c-Fluc under *lsd90* promoter (Figure 2A; Table 1). The growth rate of culture expressing luciferase under the *adh1* promoter (Figure 3B) and the *nmt1* promoter (Figure 4B) was almost similar to that of culture harbouring control vectors pART1 and pREP3X, respectively, indicating no deleterious effect of luciferase expression on host cells' growth (Figure 4B).

#### Kinetics of luciferase expression under *AOX1* promoter in *P. pastoris*

**Figure 5:**
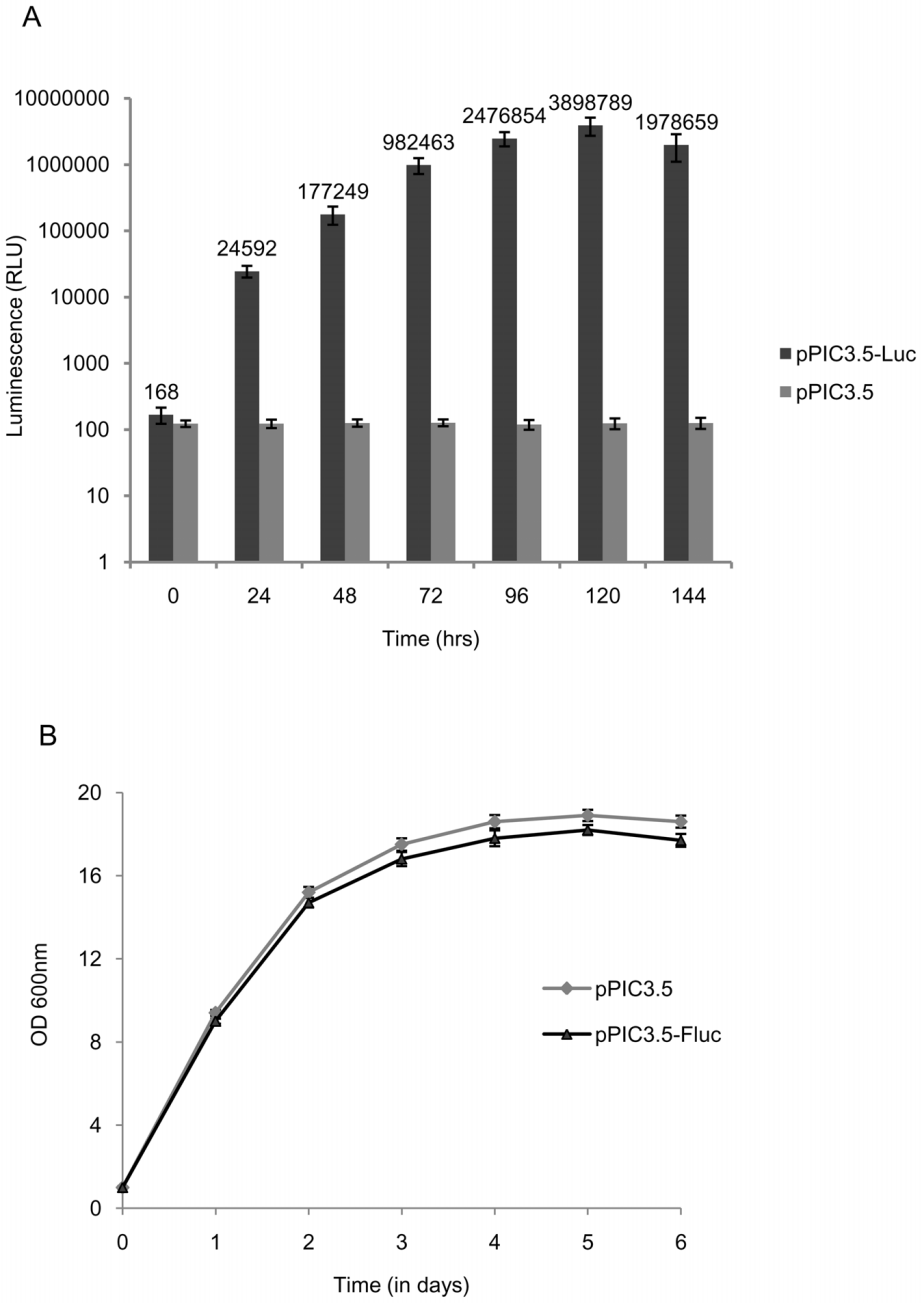
Time course of luciferase expression under control of the *AOX1* promoter of *P. pastoris*. Cultures of recombinant strain *GS115* harboring the *Fluc*-containing expression vector pPIC3.5-Fluc and the control vector pPIC3.5 were grown in suitable medium up to six days. Luciferase assay was performed in triplicate and activity was plotted against indicated time points.

For comparison, we also studied the kinetics of expression of luciferase under control of the methanol-inducible *AOX1* promoter in the vector pPIC3.5-Fluc in *P. pastoris*. A continuous increase of luciferase activity was recorded, reaching maximum level of 2.5×10^−15^ moles [(∼0.25 ng)/100 ng protein] after 5 days of induction. The luciferase activity decreased to almost half of the maximum level after 6 days of induction [1.2×10^−15^ moles; ∼0.12 ng/100 ng protein] (Figure 5). In comparison, the maximum level of luciferase expression was obtained with *lsd90* promoter after 48–64 hrs (Figure 2A). This level was at least 10-fold higher than that the maximum level of luciferase expressed achieved under the control of *AOX1* promoter after 120 hrs of induction in *P. pastoris* (Table 1).

#### Measurement of Luciferase Mrna

**Figure 6:**
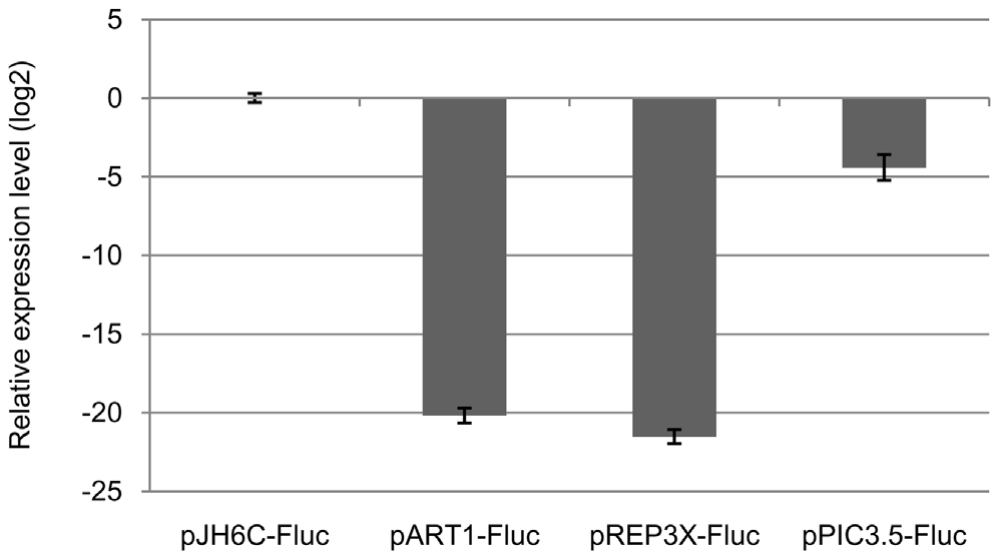
Relative-qPCR analysis of Luciferase mRNA. RNA isolated form cells harvested at time points showing maximum level of Fluc RNA under the control of different vectors was subjected to real time RTPCR analysis. Samples were analyzed in triplicate and displayed as histogram. The relative Fluc/*act1* RNA levels expressed by different vectors are displayed after normalization with respect to the vector pJH6c-Fluc. The time points were: pJH6c-Fluc: 32 hrs; pART1-Fluc: 48 hrs; pREP3X-Fluc: 16 hrs, pPIC3.5-Fluc: 96 hrs.

The level of expression of luciferase mRNA was measured by RT-qPCR and normalized against internal control housekeeping gene *act1* to determined the value of ΔC_T_ for each Fluc transcript at selected time points. Relative expression fold change of transcripts with respect to pJH6c-Fluc was quantified by ΔΔC_T_ method using 2^−ΔΔCT^ calculation [25]. As shown in Fig. 6, consistent with level of protein expression, highest level of Fluc transcript was obtained with the vector pJH6c-Fluc (32 hrs), followed by the Pichia vector pPIC3.5-Fluc (96 hrs), and pombe vectors pART1-Fluc (48 hrs) and pREP3-Fluc (16 hrs). Surprisingly, in contrast with the relative levels of Fluc protein, the levels of Fluc mRNAs were relatively much lower in case of pREP3-Fluc, pART1-Fluc as compared to pJH6c-Fluc (Figure 6, compare with Table 1). Furthermore, while Fluc protein expression with pREP3-Fluc was about twice as high as compared to that obtained with pART1-Fluc (Table 1), the mRNA expression level by these vectors are somewhat similar. The cause of this discrepancy is not known.

## Discussion

Although we selected putative promoter elements based on the high level of induction of mRNA from the linked gene sequences in response to heat and oxidative stress [24], we were surprised to observe that the promoter elements *Psp1* and *Psp2* failed to elicit significant expression of luciferase, both with and without stress conditions, in *S. pombe*. On the other hand, *lsd90* promoter (*Plsd90)* elicited high level constitutive expression of luciferase, which was not stimulated further by heat stress, while the *lsd90* gene is known to be induced by heat stress [24]. In a recent report, the promoter region of the heat-inducible gene *hsp16* has been shown to elicit induction of the GFP reporter in response to heat stress [26]. While a 0.6 kb upstream region of *hsp16* gene elicited constitutive expression of the GFP reporter, longer upstream regions of 1.2 and 1.8 kb imparted heat stress inducible expression [26]. In the present study, the lack of temperature regulation of *lsd90* promoter is surprising. Possibly, some sequence elements of *lsd90* promoter located further upstream to the region used in this study may impart the heat-inducibility. Alternatively, the promoter region used here may contain elements that respond negatively to heat stress and thus mask the heat stress-dependent induction. It is also possible that the high copy vector may titrate out the negatively acting regulator of the *lsd90* promoter in absence of heat stress. All these possibilities will be tested in future studies.

Surprisingly, cells expressing luciferase under the control of the *lsd90* promoter grew at faster rate and achieved higher OD_600_ as well as cell density than cells containing vector alone. It is possible that host cells having gene insertion in the backbone vector pJH5 [23] may impart some growth advantage. Although the exact mechanism of this characteristic is not known, it would serve as an advantageous feature for heterologous gene expression.

Here, it is pertinent to evaluate the contribution of different elements of the vector pJH6c towards the overall yield of proteins. The vector pJH5, containing the truncated *S. cerevisiae URA3m* as a selectable marker and the *mat2P RF* as *ARS* element, as a part of the backbone of the vector pJH6c, occurs at a higher copy number (∼200 copies/cell), and has greater mitotic stability (∼1.5 fold) as well as greater loss rate (∼1.25 fold) than the vector pJH2 [23], which is similar to pART1 and pREP3 (having LEU2 as a selectable marker and replication origin *ars1*(23; data not shown). The latter two vectors are, however, present at lower copy number of 50 copies/cell [23]. While a higher plasmid copy number does not necessarily cause proportionate increase in the level of expression, even assuming a linear correlation between the copy number and expression level, a cumulative contribution of plasmid copy number, plasmid stability and loss rate (4×1.5/1.25) yields a theoretically ∼4.8 fold advantage of the backbone of the vector pJH5 over pART1 and pREP3X for protein expression level. Even after accounting for this contributory factor, the *lsd90* promoter provides at least ∼4- and 8-fold higher expression level per plasmid copy than that provided by vectors containing *adh1* and *nmt1* promoters of *S. pombe*, respectively.

In conclusion, this study has led to the construction of a potentially powerful expression vector harbouring a strong constitutive *lsd90* promoter region in *S. pombe*, which can yield expression level 19- and 39-fold higher than known strong promoters of *S. pombe*, namely *nmt1* and *adh1*, respectively (Table 1). Most importantly, under shake flask conditions, it yields nearly 10-fold higher expression of luciferase after 48 hrs than that achieved with the *AOX1* promoter in *P. pastoris* after 120 hrs of culture (Table 1). After accounting for the time factor of 2.5- fold faster expression, the new expression vector shows 25-fold greater productivity of the *lsd90* promoter based vector in *S. pombe* as compared to the *AOX1* promoter in *P. pastoris*.

Based on its high level of expression, the *lsd90* promoter based vector pJH6c has great potential for commercial application. Further development of the system will involve optimization of secretion of protein by inclusion of secretory signals in the vector [27], use of protease deficient mutants [28] and addition of inexpensive chemicals like dextran sulphate to the culture medium [29]. Use of simple medium and expression regime further obviate the need for methanol or other inducers and the associated special fermenter design, also making the process more environment-friendly. Thus, the new expression vector could prove to be a viable, cost-effective and both user and environment-friendly alternative to the expression under the control of the *AOX1* promoter in *P. pastoris* for commercial scale expression of heterologous proteins of therapeutic and industrial interest in *S. pombe*.

## Materials and Methods

### Strains and media

*E.coli* strain Top10F' (Invitrogen, USA), *S. pombe* wild type strain *SPJ25* (*Msmto leu1-32 ura4D18 ade6-210 his2^−^*) and *P. pastoris* host strain *GS115* (His^−^ Mut^+^ phenotype) were used in this study. Yeast growth medium (YEA) and selective minimal medium (PMA) supplemented with appropriate amino acids [30], *P. pastoris* media (Pichia Expression Kit, Invitrogen, USA) and bacterial LB medium [31] were used for growth and maintenance of cultures.

### Cloning of the promoters of *S. pombe*

For cloning the promoters of *SPAC1F8.02C, SPBC24C6.09C* and *lsd90* genes of *S. pombe*, PCR primers (Table 2) were designed to amplify 5′-upstream region of about 1.5 Kb, 1.2 Kb and 1 Kb, respectively. Primers used for amplification of the promoters were as follows: for *Psp1*, SP1 and ASP1; for *Psp2*, SP2 and ASP2; for *Plsd90*, SP3 and ASP3. The 5′- and the 3′- primers contain Sph1 and NdeI restriction sites, respectively. The PCR products were digested with these restriction endonucleases and inserted into the same sites in the vector pJH5 [23]. The resulting vectors were named as pJH6a, pJH6b and pJH6c, respectively.

**Table 2:** **P**rimers used in this study.

Primer Name	Sequence
SP1	5′ ATGCGCATGCTGAAAGTGTGTACTGTTCGTC 3′
ASP1	5′ ATGCCATATGAGCTACTTAATTTTAAGCAATTAG 3′
SP2	5′ ATCGGCATGCAGTTGCTAAAATGGATCATAGTG 3′
ASP2	5′ ATGCCATATGATTGAAGAGGAATGTTTTTATAAT 3′
SP3	5′ ATGCGCATGCTGCTACGCTCACACTCACC 3′
ASP3	5′ ATGCCATATGGATGATGAAGAATAGAAGAATGT 3′
SP4	5′ TCGACATATGGAAGACGCCAAAAACATAA 3′
ASP4	5′ TCAGGGATCCTCACAATTTGGACTTTCCGCC 3′
SP5	5′ AGTCCTGCAGATGGAAGACGCCAAAAACATAA 3′
SP6	5′ GTCCTCGAGATGGAAGACGCCAAAAACATAA 3′
SP7	5′ AGTCGGATCCATGGAAGACGCCAAAAACATAA 3′
Luciferase For	5′ GCCAAAAGCACTCTGATTGA 3′
Luciferase Rev	5′ CACAACCTTCGCTTCAAAAA 3′
Act1-For	5′ GGATTCCTACGTTGGTGATGAA 3′G
Act1-Rev	5′ AGCAAGGGTGCTCCTCAGGAG 3′

### Construction of luciferase (Fluc) reporter plasmids

The 1653 bp Firefly (*Photinus pyralis*) luciferase gene (*Fluc*) was PCR-amplified using primers (Table 2) and cloned into the pGL3-basic vector (Promega Corp., Madison, WI). The PCR product (*Fluc*) with 5′-NdeI and 3′-BamHI sites (using primers SP4 and ASP4) was cloned downstream of the promoters *Psp1*, *Psp2* and *Plsd90* to construct vectors pJH6a-Fluc, pJH6b-Fluc and pJH6c-Fluc, respectively.

The PCR product (*Fluc*) with 5′-PstI and 3′-BamHI sites (using primers SP5 and ASP4) was cloned downstream of promoter *adh1* in vector pART1 [32] to yield the vector pART1-Fluc. Similarly, PCR-amplified product with 5′-XhoI and 3′-BamHI sites (using primers SP6 and ASP4) was cloned downstream of *nmt1* promoter in vector pREP3X [17] to construct the vector pREP3X-Fluc. Furthermore, the PCR-amplified product with 5′- and 3′-BamHI sites (using primers SP7 and ASP4) was cloned downstream of the *AOX1* promoter in the integrative vector pPIC3.5 of *P. pastoris* (Pichia Expression Kit, Invitrogen, USA) to yield the vector pPIC3.5-Fluc.

### Transformation and selection in *S. pombe*

The *Fluc* containing vectors (pJH6a-Fluc, pJH6b-Fluc, pJH6c-Fluc, pART1-Fluc and pREP3X-Fluc) described above were transformed into *S. pombe* wild type strain *SPJ25* having *leu1-32* and *ura4D18* mutations (genotype: *mat1Msmt0, leu1-32, ura4D18, his2, ade6-210*), by lithium acetate method [30]. Transformants were confirmed for presence of *Fluc*-gene insert by colony PCR using gene-specific primers.

### Transformation and selection in *P. pastoris*

*P. pastoris* transformation and selection was done according to instruction manual (Pichia Expression Kit, Invitrogen, USA). The *Fluc*-expression vector pPIC3.5-Fluc described above and the control vector pPIC3.5 were linearized with SalI and transformed into *P. pastoris* host strain *GS115* (His^−^ Mut^+^ phenotype) by lithium chloride method. Transformants were selected on minimal plates lacking histidine and screened for Mut^+^ phenotype. *Pichia* transformants were confirmed by colony PCR using gene-specific primers.

### Expression of Fluc gene in *S. pombe*

*S. pombe* strains harboring recombinant expression vectors were inoculated in selective *pombe* minimal media (PMA ura^−^ or leu^−^) and cultures were grown for 16–18 hrs at 30°C and 200 rpm. In case of the vector containing the *nmt1* promoter, thiamine was added at 25 µM final concentration to repress the promoter during growth phase. This culture was inoculated into fresh selective minimal media to a final OD_600_ of 0.1 and grown at 30°C and 200 rpm. For protein extraction and growth kinetics, samples from the culture were harvested at the indicated time points. In case of the *nmt1* promoter, secondary culture was grown up to mid-log phase (OD_600_ 0.4–0.6) in PMA leu^−^medium containing 25 µM thiamine. This culture was centrifuged and washed thrice with the same media without thiamine at room temperature. Cell pellet was resuspended in fresh PMA-leu media and grown further at 30°C and 200 rpm. Samples were harvested at specific time intervals to study growth kinetics and expression of luciferase.

All measurements of each time point were carried out in triplicate.

### Expression of Fluc gene in *P. pastoris*

Optimization and scale-up of recombinant protein expression in *Pichia* was done according to instruction manual (Pichia Expression Kit, Invitrogen, USA). A single colony of the recombinant *GS115* strain of *P. pastoris* harboring the *Fluc* gene, cloned under *AOX1* promoter was inoculated into 25 ml of BMG (Buffered Minimal Glycerol) medium in a 250 ml baffled flask and grown at 30°C in a shaker-incubator at 250 rpm until the culture reached log phase with OD_600_ of 2–6 (∼16–18 hrs). The culture was harvested by centrifugation at 3000×g for 5 minutes at RT. The supernatant was discarded and cell pellet was resuspended in 100 ml BMM (Buffered Minimal Methanol) for induction in a 1L baffled flask and grown further at 30°C with shaking at 250 rpm. To this culture, 100% methanol was added after 24 hrs intervals to give a final concentration of 1% to maintain the induction. 1 ml of culture was harvested after 24 hrs intervals for protein extraction.

All measurements of each time point were carried out in triplicate.

### Protein extraction from yeast strains

Cell extracts of *S. pombe* cultures were prepared as described previously [10]. Cell extracts of *P. pastoris* were prepared as described in Kit manual (Pichia Expression Kit, Invitrogen, USA). In case of *P. pastoris*, cells lysis was done as described for *S. pombe*, except that less number of cycles (5–6) in bead beater (Biospec, USA) were sufficient for cell breakage.

### Luminescence assay

The level of expression of luciferase was determined with Luciferase Assay System (Promega Corp., Madison, WI). All reagents were prepared as described by the manufacturer. We directly used 100 ng of cell extract for luciferase assay in place of cell lysate prepared with passive lysis buffer. All measurements were performed with GloMax™ 20/20 luminometer. Protein concentration of luciferase (moles/reaction) was determined against the calibrated standard curve, plotted between known amounts of purified recombinant luciferase (Promega Corp., Madison, WI) in moles and the respective luminescence values in Relative Light Units (RLU).

### Real-Time quantitative polymerase chain reaction (RT-qPCR)

Two-step RT-qPCR was performed to assess the relative expression of transcripts. Total RNA of cells for different time points was isolated using phenol/chloroform extraction method [33] and treated with DNase1. cDNA was synthesized from the total RNA using the cDNA Reverse Transcription Kit (Thermo scientific) in accordance with manufacturer's instructions. Maxima SYBR Green/Fluorescein qPCR Master Mix (Thermo scientific), specific forward and reverse primers of target (luciferase) and internal control (*act1*) genes were used in PCR reactions and RT-qPCR was performed in Mastercycler ep realplex Real-time PCR System. The parameters of thermocycling consisted of an initial denaturation at 95°C for 3 minute, followed by 40 cycles of denaturation (95°C/1 minute), annealing (55°C/30 second), and extension (72°C/1 minute), melting-curve analysis was carried out starting from the initial temperature 50°C to 95°C, with gradual increase of 0.5°C/15 second. The generated C_T_ values of target gene were normalized to the C_T_ value of *act1* gene and the relative fold expression changes were estimated by ΔΔC_T_ method [25]. RT-qPCR experiments were performed in triplicates and on three different days.
